# T Cell Proliferation Is Induced by Chronically TLR2-Stimulated Gingival Fibroblasts or Monocytes

**DOI:** 10.3390/ijms20246134

**Published:** 2019-12-05

**Authors:** Carolyn G. J. Moonen, Gerasimos D. Karlis, Ton Schoenmaker, Tim Forouzanfar, Bruno G. Loos, Teun J. de Vries

**Affiliations:** 1 Department of Periodontology, Academic Centre for Dentistry Amsterdam (ACTA), University of Amsterdam and Vrije Universiteit, 1081 LA Amsterdam, The Netherlands; cmoonen@uottawa.ca (C.G.J.M.); g.karlis@acta.nl (G.D.K.); t.schoenmaker@acta.nl (T.S.); b.loos@acta.nl (B.G.L.); 2 Department of Biochemistry, Microbiology and Immunology, University of Ottawa, Ottawa, ON K1H 8M5, Canada; 3 Department of Oral and Maxillofacial Surgery/Pathology, Amsterdam UMC, Vrije Universiteit Amsterdam, 1081 HV Amsterdam, The Netherlands; t.forouzanfar@acta.nl; 4 Academic Centre for Dentistry Amsterdam, University of Amsterdam and Vrije Universiteit, 1081 LA Amsterdam, The Netherlands

**Keywords:** chronic inflammation, toll-like receptor 4, leukocytes, proliferation, peripheral blood lymphocytes

## Abstract

During inflammation of the gums, resident cells of the periodontium, gingival fibroblasts (GFs), interact with heterogeneous cell populations of the innate and adaptive immune system that play a crucial role in protecting the host from pathogenic infectious agents. We investigated the effects of chronic inflammation, by exposing peripheral blood mononuclear cells (PBMCs), peripheral blood lymphocyte (PBL) cultures, and GF–PBMC cocultures to Toll-like receptor 2 (TLR2) and TLR4 activators for 21 days and assessed whether this influenced leukocyte retention, survival, and proliferation. Chronic stimulation of PBMC–GF cocultures with TLR2 and TLR4 agonists induced a reduction of NK (CD56+CD3−), T (CD3+), and B (CD19+) cells, whereas the number of TLR-expressing monocytes were unaffected. TLR2 agonists doubled the T cell proliferation, likely of a selective population, given the net decrease of T cells. Subsequent chronic exposure experiments without GF, using PBMC and PBL cultures, showed a significantly (*p* < 0.0001) increased proinflammatory cytokine production of TNF-α and IL-1β up to 21 days only in TLR2-activated PBMC with concomitant T cell proliferation, suggesting a role for monocytes. In conclusion, chronic TLR activation mediates the shift in cell populations during infection. Particularly, TLR2 activators play an important role in T cell proliferation and proinflammatory cytokine production by monocytes, suggesting that TLR2 activation represents a bridge between innate and adaptive immunity.

## 1. Introduction

Periodontitis is a multifactorial, chronic inflammatory disease initiated by both the innate inflammatory and adaptive immune system in response to pathogenic microbiota in the subgingival plaque biofilm. These aberrant responses result in the destruction of tooth-surrounding tissues by bone-resorbing osteoclasts, leading to alveolar bone loss, which could ultimately lead to tooth loss [1].

In general, innate and adaptive immune responses play a crucial role in protecting the host from pathogenic infectious agents. Transforming growth factor β and vascular endothelial growth factor may have an orchestrating role in regulating the immune response in vivo [2]. Primary responses to pathogens are initiated by pattern recognition receptors that bind to pathogen-associated molecular patterns (PAMPs) expressed on micro-organisms such as lipoproteins, lipopolysaccharides (LPS), flagellins, and microbial nucleic acids [3]. A subclass of pathogen recognition receptors (PRRs) is the family of Toll-like receptors (TLRs), which sense and recognize PAMPs on the cellular surfaces. Pathogens present in the dental plaque biofilm are recognized by TLRs on the host cells, which subsequently prompt a host immune response [4]. Among the 10 human TLRs identified so far, TLR2 and TLR4 are the most defined members. It has been shown that the cellular expression of all TLRs (apart from TLR7 and TLR8) differs significantly between healthy controls and periodontitis patients, implying a contribution in periodontitis’ pathogenesis [5]. Specifically, gingival fibroblasts (GFs), present in the alveolar bone-lining mucosa, express TLR2 and TLR4 [6,7]. TLR2 recognizes a variety of different bacterial cell components such as peptidoglycan and lipoproteins. TLR4 primarily recognizes lipopolysaccharides (LPS) of Gram-negative periodontopathogenic bacteria, including *Porphyromonas gingivalis* (*Pg*), and it acts in cooperation with several lipid and protein components such as LPS and cluster of differentiation (CD) 14 that are expressed on a variety of immune cells like monocytes, macrophages [8], and GFs [5].

At the inflamed gingival barrier, a predominance of T cells is present as part of the adaptive immune cell network [9]. The infiltration of CD3+ T cells is likely initiated by a pathogen–innate immune cell interaction, which may lead to the activation, proliferation, and differentiation of peripheral blood lymphocytes (PBLs) [10]. This is accompanied by the increased production of proinflammatory cytokines such as tumor necrosis factor alpha (TNF-α) and interleukin 1 beta (IL-1β). However, the excessive production of proinflammatory cytokines resulting from chronic stimulation of TLRs may lead to tissue destruction as apparent in periodontitis.

We recently showed that GFs play a key role in the recruitment and survival of leukocytes. In particular, GFs are essential for the proliferation of CD3+ T cells, since proliferation takes place both in GF cocultures with peripheral blood mononuclear cells (PBMCs) as well as in GF–PBL (devoid of monocytes) cocultures. Proliferation was minimal in PBMC and PBL cultures [11]. The latter study was conducted mimicking the steady-state, in other words, without taking the inflammatory component into consideration. In the present study, we further investigated our previous findings by incorporating chronic exposure to TLR2 and TLR4 activators. To date, it is unclear whether GFs play a role in the interactions between TLR activators (found on periodontal pathogens) with immune cells and how this interaction can affect number and skewness in the distribution of immune cells [12]. Furthermore, it is unknown which TLR activation is dominant in chronic inflammation. We hypothesized that chronic exposure to TLR agonists in the presence of GF may modulate an inflammatory response by interfering with the cellular distribution of leukocytes by affecting the proliferation of defined subsets of leukocytes. Therefore, the overall goal of the present study was to investigate the effects of chronic exposure to TLR2 and TLR4 agonists on the inflammatory adaptive immune response, mimicking an inflammatory environment such as apparent in chronic periodontitis. More specifically, the effects were studied through their interactions in the presence and absence of GF. Secondly, as especially monocytes are known to interact with TLR agonists, the effects of TLR agonists were studied separately in PBMC and PBL cultures. In order to mimic an enduring bacterial pressure, as is the case during chronic periodontitis, TLR2, TLR4, or a combination of both agonists, were added to the PBMC/PBL cultures and GF cocultures for 21 days, where after the effects on leukocyte survival and selective proliferation were assessed. The aim of the present study, therefore, was to analyze the effect of periodontitis-relevant agonists on proliferation of PBMCs, where specifically the roles of GFs and monocytes were assessed. We hypothesize that bacterial products as represented by TLR agonists influence the proliferation of specific subsets within peripheral blood.

## 2. Results

### 2.1. TLR Activation Reduces PBMC Survival in GF-PBMC Cocultures

**Figure 1:**
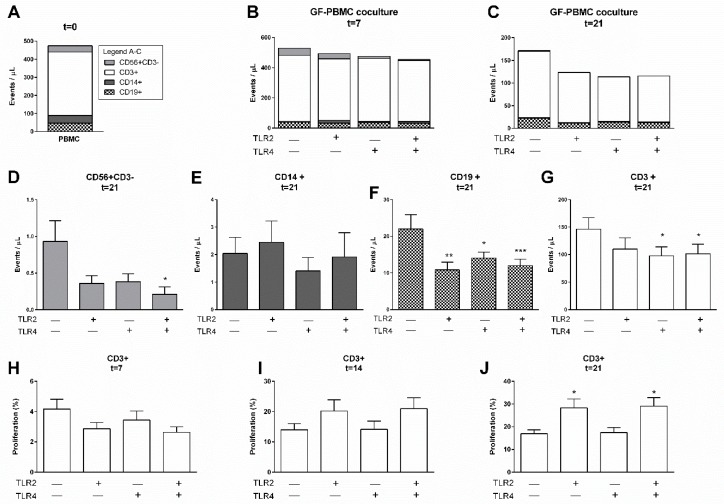
TLR agonists affect the survival of PBMCs in the presence of gingival fibroblasts. Characterization of heterogeneous cell populations: (**A**) immediately after isolation (t = 0), (**B**) after 7 days, and (**C**) after 21 days. No significant differences were found between total leukocyte cell numbers of control, TLR2, TLR4, and TLR2+TLR4 stimulated conditions for 7 nor for 21 days. Specification of cells from (**C**) at 21 days is shown for (**D**) CD56+CD3−, (**E**) CD14+, (**F**) CD19+, and (**G**) CD3+ cells, showing diminished numbers of CD56+, CD19+, and CD3+ cells and not CD14+ after TLR2 or TLR4 stimulation. The proliferation of CD3+ cells over time is presented as percentage divided cells at (**H**) 7, (**I**) 14, and (**J**) 21 days. Over time, a trend of increased proliferation was seen (note differences in values at *y*-axes). A significant increase in proliferation of CD3+ cells was seen in PBMC–GF cocultures with TLR2 agonists, after 21 days. Data are presented as events per microliter (**A**–**G**), + SEM (**D**–**G**), or in percentages + SEM (**H**–**J**). *n* = 6 GF donors. Statistical significance was calculated (**D**–**J**) using an ANOVA with multiple comparisons (Tukey post-hoc). Significant differences are shown in comparison to control conditions (without TLR agonists). * *p* < 0.05, ** *p* < 0.01, *** *p* < 0.001.

In the present study, we mimicked the effect of chronic inflammation on the survival and retention of PBMCs by investigating the stimulatory additional effects of TLR2, TLR4, or a combination of stimulation of both receptors. Flow cytometric measurements are presented as events per microliter (µL) of the live-gated population (Gate P1, Supplementary Figure S1A), based on forward/sideward scatter properties, where CD56+CD3− (NK cells), CD3+ (T cells), CD14+ (monocytes), and CD19+ (B cells) cells were identified. The employed gating strategy is presented in Supplementary Figure S1. The heterogeneous cell composition of GF–PBMC cocultures is illustrated in Figure 1, before culturing (t = 0, Figure 1A), at 7 days (Figure 1B), and at 21 days (Figure 1C), cultured with or without the specified TLR agonist. The CD3+ cell population was consistently present in all conditions and at all time points (white bars). With these results, we confirm previous findings that GFs mediate the survival and retention of PBLs over the culture period of 21 days (control conditions, Figure 1B,C). Interestingly, when assessing the separate cell populations at the final time point of 21 days, significantly fewer CD56+CD3− (NK cells, Figure 1D), CD19+ (B cells, Figure 1F), and CD3+ (T cells, Figure 1G) cells were found in the presence of TLR agonists, either when a single agonist was present or in a combination of both agonists (TLR2 and TLR4), in comparison to control conditions. The addition of TLR agonists for 21 days did not affect the number of monocytes (CD14+ cells) over time (Figure 1E).

### 2.2. TLR2 Agonists Induce T Cell Proliferation in the Presence of GF

The above results show a marked decreased survival of most leukocyte subsets after chronic exposure to TLR2 and TLR4 agonists. Since TLR activation is associated with the activation and differentiation of T cells [10,12,13], we next investigated whether these lymphocytes may have proliferated as well. Accordingly, PBMCs and monocyte-depleted PBMCs (i.e., PBLs) were labeled with carboxyfluorescein succinimidyl ester (CFSE) before culturing, after which proliferation was assessed after 7, (Figure 1H), 14 (Figure 1I), and 21 days (Figure 1J). The employed gating strategy for T cell proliferation quantification is presented in Supplementary Figure S2. Previously, we reported a selective proliferative effect of CD3+ cells mediated by GFs [11]. Here, we confirm these findings (control conditions), shown as increased percentages over time going from roughly 4% at 7 days to 16% at 21 days (Figure 1H,J). No proliferative effect by TLR agonists was observed either at 7 (Figure 1H) or 14 days (Figure 1I). However, after 21 days, significantly more proliferation was observed in coculture conditions with TLR2 agonists (Figure 1J). Thus, GFs can induce T cell proliferation and was significantly increased in the presence of TLR2 agonist after 21 days. No proliferative effect on CD19+ and CD56+CD3− cells in the presence of GF was observed in the control conditions or in the presence of TLR2 or TLR4 agonists (Supplementary Table S1).

### 2.3. PBMCs Express Significantly More TLR2 and TLR4 than GFs

**Figure 2:**
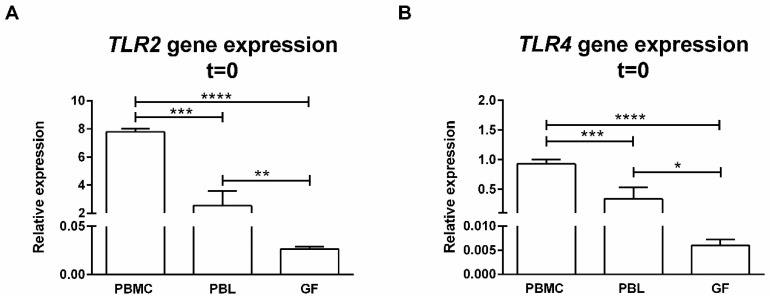
TLR2 and TLR4 are expressed by PBMC, PBL, and GF cultures. Relative gene expressions of (**A**) *TLR2* and (**B**) *TLR4* in freshly isolated PBMCs, PBLs, and GFs (passage 5). Both PBMCs and PBLs showed higher *TLR2* and *TLR4* gene expression than GFs. Accordingly, PBMCs expressed more *TLR2* and *TLR4* than PBLs. Both genes are expressed relative to the mean of the housekeeping gene *HPRT*. Primer sequences are provided in Table 1. Data are presented as means + standard deviations. *n* = 2 buffy coats, *n* = 6 GF donors. Statistical significance was calculated using an ANOVA with multiple comparisons (Tukey post-hoc). * *p* < 0.05, ** *p* < 0.01, *** *p* < 0.001, **** *p* < 0.0001.

It has been previously reported that GFs express TLRs [6]. Here, we confirmed the gene expressions of TLR2 (Figure 2A) and TLR4 (Figure 2B) by GFs (*n* = 6), which were used in our coculture experiments. The expressions of TLR2 and TLR4 of unstimulated PBMCs were significantly higher (*p* < 0.0001) than that of GFs when comparing GF expression to that of PBMCs and PBLs. We compared the TLR2 and TLR4 expressions of PBMCs with monocyte-depleted PBMCs (PBLs) to identify the role of monocytes in TLR expression. A significantly lower expression of TLR2 and TLR4 was observed in PBLs compared to PBMCs. The majority of TLR2 and TLR4 expressions can be attributed to monocytes since PBMCs comprise approximately 20% monocytes.

Therefore, it is possible that chronically TLR2-stimulated T cell proliferation could be (partially) attributed to monocytes that were present in the GF–PBMC cocultures. To assess this in more detail, we next investigated whether activation of TLR2 and TLR4 would have an impact on PBMCs alone or on monocyte-depleted PBMCs (PBLs).

### 2.4. Monocytes Play an Important Role in PBL Survival in the Absence of GFs

**Figure 3:**
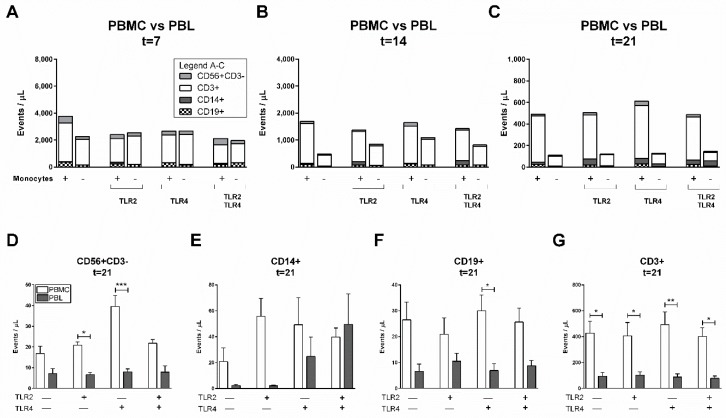
Monocytes play a role in the retention of PBLs and is not affected by TLR agonists. Characterization of heterogeneous cell populations: (**A**) after 7, (**B**) after 14, and (**C**) after 21 days. The different culture conditions are presented on *x*-axes (**A**–**C**), with or without monocytes, and in the presence or absence of TLR agonists. Fewer cells retain over time (note differences in values at *y*-axes) and in PBL conditions (without monocytes). Specification of cells from (**C**) (at 21 days) are shown for (**D**) CD56+CD3− (NK cells), (**E**) CD14+ cells (monocytes), (**F**) CD19+ (B cells), and (**G**) CD3+ (T cells). No significant differences were found between cell compositions of control, TLR2, TLR4, and TLR2+TLR4 conditions (calculated using an ANOVA with multiple comparisons, Tukey post-hoc). Data are presented as events per microliter, *n* = 2 buffy coats. Significant differences between PBMC and PBL cultures were calculated (**D**–**G**) using paired *t*-tests. * *p* < 0.05, ** *p* < 0.01, *** *p* < 0.001.

We confirmed the pivotal role GFs play in the survival and selective proliferation of PBLs. In this system, proliferation was likely mediated at least in part by GFs, considering that we previously showed that there is no difference in T cell proliferation when PBMCs (which contain monocytes) or PBLs were added to GFs. However, monocytes, more than GFs, play an important role as a primary target cell for bacterial recognition and killing as they express high levels of TLR2 and TLR4 (Figure 2). As such, we next investigated whether TLR2, TLR4, or a combination of both agonists would affect cell heterogeneity in the absence of GFs (Figure 3A–C). Additionally, the role of monocytes in PBL survival was investigated by comparing PBMCs with monocyte-depleted PBMCs (PBLs). Though not apparent at early time points of 7 (Figure 3A) and 14 days (Figure 3B), clearly more cells survived in the presence of monocytes independent of the presence of TLR agonists at 21 days (Figure 3C). In the presence of TLR4 agonists in PBMC cultures, more CD56+CD3− cells survived (Figure 3D). When further assessing the separate cell populations at the final time point (Figure 3C), no differences in the numbers of CD14+ (Figure 3E), CD19+ (Figure 3F), and CD3+ (Figure 3G) populations were observed in the presence of TLR agonists in comparison to control conditions. This is clearly different from the GF–PBMC cultures, where TLR agonists caused a decline in leukocyte numbers (Figure 1D–G).

Interestingly, monocytes improved the survival rates of PBLs after 21 days (Figure 3C). Accordingly, more PBLs were observed in conditions with monocytes (PBMC conditions; white bars, Figure 3D,F,G), regardless of the presence of TLR agonists. This and previous results suggest that, in addition to GFs, monocytes also play a key role in the survival and retention of PBLs.

### 2.5. TLR2 Induces T Cell Proliferation in the Presence of Monocytes

**Figure 4:**
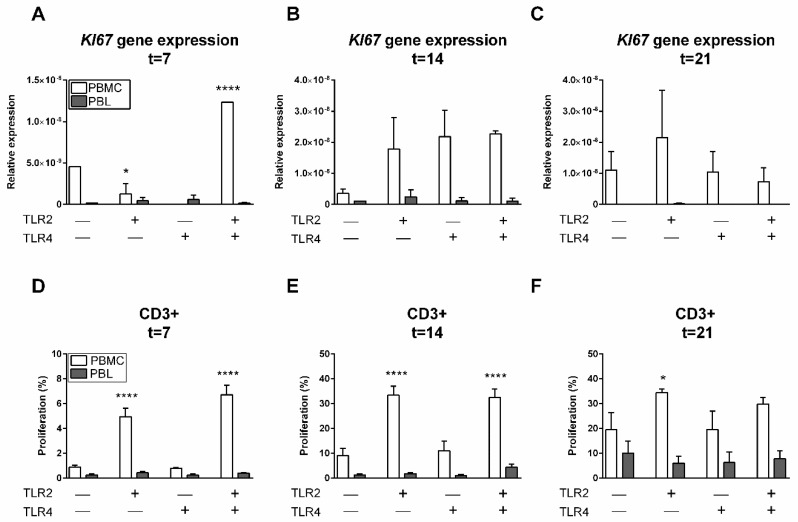
TLR2 induces T cell proliferation in the presence of monocytes. Relative gene expression of proliferation marker *KI67* after 7 (**A**), 14 (**B**), and 21 days (**C**) was increased in PBMC cultures in comparison to PBL cultures. After 7 days, the relative gene expression of *KI67* was significantly increased in PBMCs in the presence of TLR2 and TLR4 agonists. Gene expression is relative to the mean expression of the housekeeping gene *HPRT*. Quantification of CD3+ proliferation after (**D**) 7, (**E**) 14, and (**F**) 21 days. Here, PBMCs (white bars) or PBLs (grey bars) were cultured without TLR agonists (control), with TLR2, TLR4, or a combination of TLR2 and TLR4 agonists. A significant increase of CD3+ proliferation was observed in conditions containing TLR2 agonist and monocytes (PBMC cultures). Data are presented as means + standard deviation, *n* = 2 buffy coats. Significant differences are shown in comparison to control conditions (without agonists). Statistical significance was calculated using an ANOVA with multiple comparisons (Tukey post-hoc). * *p* < 0.05, ** *p* < 0.01, *** *p* < 0.001.

The results presented above suggest the importance of monocytes in the survival of PBLs, while TLR agonists did not seem to play a significant role in the retention and survival of PBLs. At gene expression levels, *KI67*, a proliferative marker [14], was investigated. At 7 days, a significant increase of *KI67* expression in PBMC cultures with TLR2 and TLR4 agonists was found (*p* < 0.0001, Figure 4A). Interestingly, a significantly lower *KI67* expression (*p* = 0.0147) was found in PBMC cultures with TLR2 agonists in comparison to control conditions. After 14 days, a trend of higher *KI67* expression was shown in PBMC cultures with TLR agonists (*p* = 0.1977, Figure 4B). In general, *KI67* was expressed solely in the presence of monocytes (PBMCs, white bars). This suggests the importance of monocyte–PBL interactions for cell proliferation in the presence of a TLR2 agonist.

Next to *KI67* gene expression as a general proliferation marker, we investigated CFSE-labeled cell proliferation with flow cytometry to identify which cells had proliferated within the heterogeneous cell population. Accordingly, PBMCs and monocyte-depleted PBMCs (PBLs) were labeled with CFSE before culturing, after which proliferation was assessed after 7, (Figure 4D), 14 (Figure 4E), and 21 days (Figure 4F). No proliferation was observed of CD19+, CD56+CD3−, or CD14+ cells at any of the aforementioned time points (data not shown); however, CD3+ cells strongly proliferated under the influence of TLR2 (Figure 4D–F).

In comparison to control conditions, significantly more divided CD3+ cells were observed in conditions where TLR2 agonists were present (Figure 4D–F) and only in PBMC cultures that contained monocytes. This proliferation was strongly induced by TLR2 agonists after 14 days (note the differences in values at the *y*-axis, Figure 4E). No synergistic or inhibiting effect of TLR4 was observed since the percentage of proliferating cells was comparable to conditions where only the TLR2 agonist was added. The proliferative effect of TLR2 remained stable after 14 and 21 days (Figure 4E,F). Interestingly, a significantly lower percentage of CD3+ cells proliferated in the absence of monocytes (PBL conditions, grey bars, Figure 4D–F), which suggests an important role of monocytes in T cell proliferation in the presence of TLR2 agonists. Altogether, this demonstrates that TLR2 agonists, in the presence of monocytes, induced T cell proliferation.

### 2.6. TLR2 Agonists Induce Proinflammatory Cytokine Production in the Presence of Monocytes

**Figure 5:**
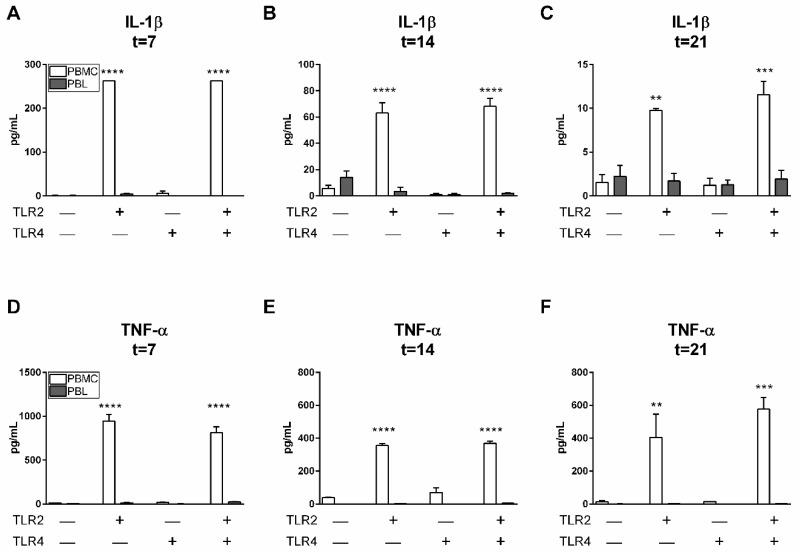
TLR2 induces proinflammatory cytokine production of IL1-β and TNF-α in the presence of monocytes. Cytokine protein levels (pg/mL, shown on *y*-axes) of interleukin 1 beta (IL-1β) and tumor necrosis factor alpha (TNF-α) after 7, 14, and 21 days. A significantly increased expression of IL-1β (**A**–**C**) and TNF-α (**D**–**F**) over the whole time period was observed in PBMC cultures in the presence of TLR2 agonists. Over time, IL-1β and TNF-α expressions decreased (note differences in values at *y*-axes). Data are presented as means + standard deviation, *n* = 2 buffy coats. Significant differences are shown in comparison to control conditions without TLR agonists. Statistical significance was calculated using an ANOVA with multiple comparisons (Tukey post-hoc). ** *p* < 0.01, *** *p* < 0.001, **** *p* < 0.0001.

Since it is known that TLR activation of monocytes may affect T cell populations through the expression of monocyte-derived inflammatory cytokines [10,15], we next investigated whether chronic TLR2 and TLR4 activation led to the induction of proinflammatory cytokine production. Protein expressions of IL-1β (Figure 5A–C) and TNF-α (Figure 5D–F) were significantly increased in the presence of monocytes (PBMC conditions, Figure 5) and TLR2 agonists. Clearly, the presence of monocytes (PBMC conditions, Figure 5), was associated with the production of IL-1β and TNF-α. When assessing the effect of TLR2 stimulation over time, different IL-1β and TNF-α production responses were observed (please note differences in *y*-axes). The presence of TLR2 agonists mediated the production of IL-1β, reducing its production strongly over time, from 262 pg/mL at 7 days to 10 pg/mL at 21 days (Figure 5A,C). Conversely, TLR2 agonist impact on the expression of TNF-α was more limited as the expression declined from 945 pg/mL to 405 pg/mL.

### 2.7. TLR2 and TLR4 Agonists Enhance GF-Mediated T Cell Proliferation in the Absence of Monocytes

**Figure 6:**
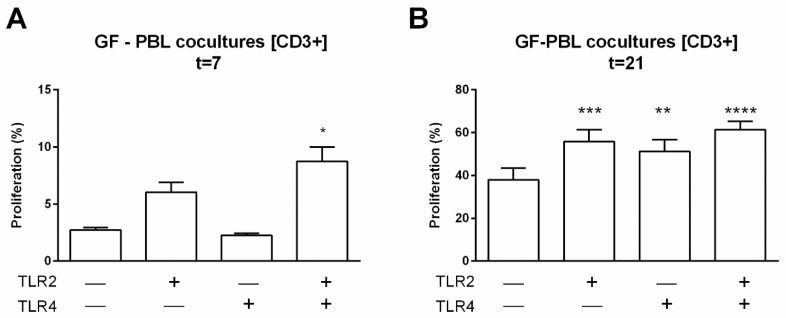
TLR agonists enhance the proliferation of T cells in the absence of monocytes. Proliferation was measured by CSFE labeling at day 7 (**A**) and day 21 (**B**). Over time, a trend of increased proliferation was seen (**A**), day 7. A significant increased proliferation of CD3+ cells was seen in PBL–GF cocultures with TLR agonists after 21 days. Data are presented in percentages + SEM. *n* = 6 GF donors. Significant differences are shown in comparison to control conditions (without TLR agonists). Statistical significance was calculated using an ANOVA with multiple comparisons (Tukey post-hoc). * *p* < 0.05, ** *p* < 0.01, *** *p* < 0.001, **** *p* < 0.0001.

All above experiments indicate that monocytes play a role in the selective proliferation of surviving T cells, both when cultured as PBMCs alone or when cocultured with GFs. We finally investigated whether GFs may also induce PBL proliferation in the absence of monocytes. To achieve this, we added cocultured GFs and PBLs in the presence of TLR agonists and analyzed proliferation of all leukocyte subsets at 7 and 21 days. Again, no proliferation of NK cells or B cells was observed under any condition. However, GF induced T cell proliferation in the absence of TLR agonists, confirming our previous findings [11]. Chronic exposure to TLR agonists induced an enhanced proliferation at 7 days when both agonists were added and at day 21, both when added separately or in combination (Figure 6). These data demonstrate that GFs are capable of inducing T cell proliferation in the absence of monocytes, an effect that is enhanced during chronic exposure with TLR agonists.

## 3. Discussion

During gingival inflammation, resident cells of the periodontium (in particular GFs), interact with heterogeneous effector cell populations of the innate and adaptive immune responses that have infiltrated the periodontal tissues from the bloodstream [16,17]. The main goals of these resident GFs and these innate and adaptive immune responses are the protection of the host from spreading pathogenic infections and the structural maintenance of the periodontal tissues. Recently, we showed that GFs play an important role in the long-term retention and survival of lymphocytes [11]. This novel role was further investigated in the current study by mimicking a chronic inflammatory model in vitro to investigate the interactions between the resident cells (GFs), bacterial products (TLR agonists), and innate and adaptive immune cells (PBMCs) on immune responses including cell survival, cell proliferation, and proinflammatory cytokine production.

Previously, we showed that a short incubation of three hours with *Pg* did not affect subsequent osteoclast formation from cocultures of PBMCs with periodontal ligament fibroblasts [18]. Here, we hypothesized that chronic activation by TLR agonists in the presence of GFs may modulate an inflammatory response by interfering with the cellular distribution of leukocytes by affecting the proliferation of defined subsets of leukocytes. We confirmed the previous findings [11] that GFs play a significant role in the long-term survival, retention, and selective proliferation of PBLs. Furthermore, the overall aim of the present study was to investigate the effects of chronic exposure to TLR2 and TLR4 agonists on the inflammatory adaptive immune response, mimicking an inflammatory environment such as apparent in chronic periodontitis. More specifically, the effects were studied through their interactions in the presence and absence of GFs. Accordingly, this study demonstrated that chronic stimulation of GF–PBMC cocultures with TLR2 and TLR4 agonists induced a reduction of CD56+CD3−, CD3+, and CD19+ cells, whereas the number of TLR-expressing monocytes was unaffected. These effects were only seen in the GF–PBMC and the GF–PBL cocultures and not in the PBMC cultures without GF. In particular, activation by TLR2 agonists stimulated CD3+ cell proliferation in the presence of GF. Since especially monocytes are known to interact with TLR agonists, the effects of TLR agonists were studied separately in PBMCs and PBLs. Here, we found that monocytes played a significant role in the survival, retention, and selective (only T cells) proliferation of PBLs, independent of TLR agonists. Therefore, it can be posited that both GFs and monocytes mediate the diversity of the cellular population at the site of periodontal inflammation. The observed T cell proliferation was induced by GFs and monocytes, especially after stimulation of the TLR2.

### 3.1. TLR2 and TLR4 Responses on GFs versus Monocytes

The innate immune response is the first line of defense in response to pathogens and comprises three subsequent events: microbial recognition, activation of signaling pathways, and activation of effector mechanisms. The first step, microbial recognition, is mainly mediated by TLRs. Several periodontopathogens have been identified, of which *Pg* is one of the bacteria in the initiation and progression of periodontitis and which interacts either via TLR2 or TLR4 [19,20,21,22].

Although it is well known that GFs express TLR2 and TLR4, we measured significantly higher *TLR2* and *TLR4* gene expression levels in freshly isolated PBMCs and PBLs. Indeed, GFs also expressed *TLR2* and *TLR4,* but this was over 150-fold lower than that in PBMCs. Reasonably, immune cells are more likely to interact with pathogens via TLRs than GFs. Accordingly, GFs interact with pathogens mostly to initiate immune responses and thereby attract immune cells to prompt the activation of effector mechanisms. However, the interaction of resident GFs with infiltrating PBMCs could possibly synergistically increase TLR expression. Our results suggest a specific role of GFs in reducing leukocyte numbers in the presence of TLR activators. Accordingly, cell numbers of a broad range of leukocytes (NK cells, B cells, and T cells) were reduced. This remarkable and novel effect was not seen in PBMC cultures, where TLR activation did not affect leukocyte numbers.

### 3.2. The Role of Monocytes in TLR Activation

Next to the role of GF, we investigated the role of monocytes in TLR expression, survival of PBLs, and T cell proliferation. The current study showed that monocytes expressed high levels of TLR, making them the key responsive cells to TLR agonists. The presence of monocytes was beneficial for the survival of PBLs, irrespective of the presence of TLR agonists. Interestingly, marked effects on cell survival were seen only after prolonged culture (21 days), a relatively late time point, often neglected in many PBMC studies. Since significantly less PBLs (T, B, and NK cells) survived in the absence of monocytes, we concluded that monocytes played a significant role in the survival of these cells.

CD14 acts as a co-receptor along with TLR4 for the detection of bacterial LPS [23] and is expressed on immune cells like monocytes and macrophages [24,25]. Studies have also shown that CD14 ([6,25], and this study) as well as *TLR2* and *TLR4* are expressed on human GFs and, thus, mediate pathogen interactions with GFs. Liu et al. (2015) exposed a monocytic cell line to a TLR4 agonist, which enhanced the expression of adherence genes *LFA-1* and *VLA-4,* suggesting that binding of monocytes to fibroblasts is likely partly regulated by LFA-1 and VLA-4 [26,27].

Interestingly, more CD14+ cells were found in cultures of PBLs, while these cultures only consisted of <5% monocytes. Since PBL cultures responded by a late increase in CD14+ cells when TLR4 agonist was added, our results suggest that TLR4 activation promotes the survival of CD14+ cells. Also, we showed that monocytes play an essential role in PBL survival and T cell proliferation under TLR2-stimulated conditions. Thus, in this system under these conditions, monocytes play a key role in innate and adaptive immune responses.

### 3.3. Proinflammatory Cytokine Production by TLR2 Activation

In addition to their pivotal role in host immune defense against invading pathogens, TLRs are capable of modulating inflammation by initiating a series of downstream signaling events that drive cellular responses, including the ones resulting in various proinflammatory cytokines such as TNF-α and IL-1β [28]. GFs continuously encounter various pathogenic compounds from bacteria that have been translocated from periodontal sites into connective tissue. Upon interaction, GFs can produce chemokines to attract an infiltrate of inflammatory cells and (proinflammatory) cytokines in order to expand the immune response and resolve microbial infections. However, excessive production of proinflammatory cytokines resulting from chronic stimulation of TLRs may eventually lead to tissue destruction. It has been previously reported that LPS-activated monocytic cells are a source of proinflammatory cytokines and chemokines, which are mediated by TLR2 and TLR4 pathways [29]. In the current study, a high concentration of the proinflammatory cytokine IL-1β was measured over time in cultures of PBMCs stimulated with TLR2. This production was significantly higher at all time points than in cultures without TLR2 agonists and without monocytes, indicating that the increased production of IL1-β originated from TLR2-activated monocytes.

TNF-α was present at high levels in PBMC cultures stimulated with TLR2 agonists and remained present for 21 days. As previously published, GF cocultures with PBMCs induce high levels of TNF-α while GFs alone do not [30]. In contrast to the latter study where TNF-α levels severely dropped from 7 days until 21 days, TNF-α remained relatively high in PBMC cultures in the current study, exclusively in the presence of chronic TLR2 agonist exposure. Together with our new findings, we suggest that cellular interactions between fibroblasts and cells from peripheral blood are needed for proinflammatory cytokine production. TLR2 activation boosts this proinflammatory induction significantly indicating that this agonist likely represents a potential bridge between innate and adaptive immunity.

### 3.4. Effects of TLRs on T Cell Activation/Proliferation

The results support our hypothesis that chronic TLR activation leads to increased T cell proliferation. In addition to driving inflammatory responses, TLRs also regulate cell proliferation and survival [31]. Cell proliferation is critical for immune cell expansion, resolution of inflammatory responses, and tissue repair or regeneration processes. While fewer cells survived in the presence of TLR agonists, selective TLR2-driven T cell proliferation was observed. Accordingly, a significant increase in T cell proliferation occurred after 21 days in the presence of GF and TLR2 agonists. The significant increase in IL-1β and TNF-α production could be partially responsible for the observed increased proliferation since it has been shown by others that these cytokines play a role in T cell proliferation [32,33]. It is generally considered that selective T cell proliferation is a consequence of antigen presentation by antigen-presenting cells. This manuscript fails to identify the presented antigens induced by TLR activation. One could consider that the observed selective T cell proliferation in conditions without TLR agonists could be due to the allogenic nature of cell interactions, at least in the cocultures. However, TLR activation consistently resulted in extra proliferation on top of this relatively high baseline proliferation in PBMC–GF, PBMC alone, and PBL–GF cultures.

## 4. Materials and Methods

### 4.1. Gingival Fibroblasts (GFs)

GFs were isolated from discarded third molars (wisdom teeth) of 6 healthy individuals. Donors did not have any overt signs of gingival inflammation and periodontitis. After tooth extraction, free gingiva and part of the interdental gingiva were still attached to each tooth. To avoid mixing with attached periodontal ligament, the gingival tissue was scraped off with a scalpel towards the coronal part of the tooth. Tissue fragments were collected and washed twice in culture medium (Dulbecco’s minimal essential medium, Gibco BRL, Paisley, Scotland) supplemented with 10% fetal calf serum (Fetal Clone I, Hyclone Laboratories, Logan, UT, USA), 1% antibiotics (100 U/mL penicillin, 100 µg/mL streptomycin, and 250 ng/mL amphotericin B; Antibiotic antimycotic solution, Sigma Aldrich, St. Louis, MO, USA). Subsequently, tissue fragments were cut into small pieces and expanded for 4 passages in a humidified atmosphere of 5% CO_2_ in ambient air at 37 °C. All experiments were performed with GFs of passage 5.

Sampling from the donors was conducted at the Department of Oral and Maxillofacial Surgery and Oral Pathology, Amsterdam University Medical Centre, location VUmc, Amsterdam, The Netherlands.

The current study was exempt from ethical approval since Dutch law at the time of tissue collection did not require ethical approval for human surgical waste material such as wisdom teeth. GFs were retrieved from healthy donors who agreed to donate the biological waste material (third molars with attached gingiva) after extraction of their wisdom teeth as part of their treatment in the Academic Center for Dentistry Amsterdam (ACTA) and stored in liquid nitrogen. Informed and written consent was obtained from all individuals, and samples were coded to guarantee the anonymity of the donors as required by Dutch law. Researchers handling the fibroblasts could not gain access to the identity of the donors.

### 4.2. Peripheral Blood Mononuclear Cell (PBMC) Isolation

PBMCs were isolated from buffy coats (*n* = 3, Sanquin, Amsterdam, The Netherlands) taken from healthy donors by standard density gradient centrifugation with Ficoll-Paque. Briefly, buffy coats were diluted 1:1 in 1% citrate solution (1.55 M sodium citrate, 0.10 M citric acid in sterile water, pH 7.4, both Merck Millipore, Darmstadt, Germany) in sterile PBS. Subsequently, 25 mL of the diluted buffy coat mixture was carefully layered by slowly pipetting the diluted buffy coat over 15 mL Lymphoprep (Axis-Shield Po CAS, Oslo, Norway). PBMCs were collected from the interphase after 30 min of centrifugation (800 RCF, no brake). Finally, after 3 washes in 1% citrate–PBS, PBMCs were recovered in culture medium and used for the subsequent experiments.

### 4.3. Peripheral Blood Lymphocyte (PBL) Isolation

PBLs were isolated from PBMCs after negative selection with CD14+ conjugated magnetic microbeads (Miltenyi Biotec, Bergisch Gladbach, Germany). The CD14 negative fraction, further referred to as PBL, was collected, washed in PBS, and recovered in culture medium. Finally, the purity of the PBLs was tested with efluor-450 conjugated anti-human CD14 (clone 61D3, BD Biosciences, Piscataway, NJ, USA) and found to be 99.4% ± 0.21% CD14− (*n* = 3, average ± SEM), as confirmed by flow cytometry (FACSverse™, BD Biosciences).

### 4.4. Cultures with TLR Agonists

A total of 500,000 PBMCs (*n* = 3 buffy coats) were cultured in duplicate alone or in coculture with GFs (15,000 per well, *n* = 6). Additionally, PBLs (400,000 per well, *n* = 2 buffy coats) were cultured in duplicate. Cultures were maintained in a humidified atmosphere of 5% CO_2_ in ambient air at 37 °C. Cultures were refreshed every 3 days. A titrated concentration of TLR2 ligand (10 ng/mL, PAM2CSK4, #14E14-MM, Invivogen, San Diego, CA, USA), TLR4 ligand (10 ng/mL, LPS-*Pg* Ultrapure, Version #14F18-MM, Invivogen), or a combination of both, was added to the culture media at the start of the experiment and with every subsequent culture media refreshment. Titration of TLR2 and TLR4 was assessed with osteoclastogenesis as read-out (G.D. Karlis, manuscript in preparation). Control conditions contained culture media without TLR agonists but included similar addition of vehicle (distilled water) than the TLR conditions.

### 4.5. Cell Population Characterization with Flow Cytometry

The heterogeneity of the cell suspensions from 3 independent experiments was characterized at 7, 14, and 21 days with flow cytometry. All leukocytes present in PBMCs were identified: CD3+ (T cells), CD19+ (B cells), CD56+CD3− (NK cells), and CD14+ (monocytes). Before the start of the experiment, PBMCs and PBLs were characterized. At 7, 14, and 21 days of culture, cells were trypsinized with 0.05% Trypsin in 0.5 mM EDTA–PBS for 15 min at 37 °C. Duplicate samples were pooled and suspended in FACS buffer (20 µg/mL sodium azide, 0.5% BSA in sterile PBS). Cell suspensions were incubated at 4 °C in the dark with a mixture of monoclonal antibodies (mAb, listed in Table 2). After 30 min of incubation, cells were washed with FACS buffer to remove remaining unbound antibodies. Finally, cells were recovered in cold FACS buffer for analysis.

Flow cytometric analysis was performed on a BD FACSverse™ flow cytometer (BD Biosciences) with a medium flow rate (63 µL/min). Quantification of cells was performed by automatic volumetric measurements over the entire acquisition time per sample. At least 10,000 cells were analyzed per sample. Optimal antibody concentrations, spillover values, and multicolor compensation settings were determined during previous experiments. These settings were subsequently applied during all experiments. Quantification of cells was performed by automatic volumetric measurements over the entire acquisition time per sample. Gating of the populations and subsequent gates were based on PBMC controls without GFs to exclude GF cells from coculture data (Supplementary Figure S1). Flow cytometry data were analyzed using the associated FACSuite™ software (Version 1.0.5, BD Biosciences).

### 4.6. Proliferation Assay

Before (co)culturing, PBMCs and PBLs were labeled with FITC-labeled Celltrace^TM^ carboxyfluorescein succinimidyl ester (CFSE, Invitrogen by Thermo Fischer, Eugene, OR, USA) for cell proliferation detection. During the labeling process, cells were handled in the dark. PBMCs (10 × 10^6^ cells/mL) were washed with 5% FCS in PBS and incubated at 37 °C with 3 µM CFSE. After 7 min of incubation, cells were washed with 5% FCS in PBS for 15 min at RT. After a final wash step, the cell pellet was suspended in culture medium and checked for labeling efficiency with flow cytometry (≥85%). Finally, CFSE-labeled cells were either cultured with or without GFs for 7, 14, and 21 days in the dark at 37 °C with 5% CO_2_. Proliferation observed through a decreasing CFSE signal, assessed as a novel cell population that had a distinctly lower CFSE signal, was detected with flow cytometry (FITC channel: excitation 492 nm, emission 517 nm) for live, CD3+ gated cells (Supplementary Figure S2). Shifts of the CFSE signal were also analyzed for CD19+, CD56+, and CD14+ cells, but no proliferation in terms of a demarcated population was observed for these cell types.

### 4.7. Gene Expression Analysis

*TLR2* and *TLR4* gene expressions of PBMCs and PBLs were analyzed immediately after isolation (t = 0, *n* = 2) and of all GFs used in coculture experiments (*n* = 6). Expression of the cellular proliferation marker *KI67* was analyzed in PBMC and PBL cultures at 7, 14, and 21 days. RNA was extracted using a commercial spin-column kit (RNeasy Mini kit, Qiagen, Düsseldorf, Germany) according to the manufacturer’s instructions. Reverse transcription was performed using an MBI Fermentas cDNA synthesis kit (Thermo Fisher Scientific). Real-time quantitative PCR (RT-qPCR) was performed with a Roche Lightcycler 480 II. The primers used for gene expression analysis are listed in Table 1. Relative gene expression was normalized by the mean expression of the housekeeping gene *hypoxanthine phosphoribosyltransferase* (*HPRT*) following the comparative cycle threshold (Ct) method. Data are presented as the mean relative fold expression (2^−∆Ct^).

### 4.8. Cytokine Production Analysis

At 7, 14, and 21 days, conditioned medium was collected from all PBMC and PBL cultures and stored at −80 °C for cytokine production analysis. Concentrations of TNF-α and IL-1β were measured with an enzyme-linked immunosorbent assay (ELISA, R&D Systems, Minneapolis, MN, USA) according to the manufacturer’s instructions.

### 4.9. Statistics

All data sets were analyzed using GraphPad Prism software (version 6.07, La Jolla, CA, USA). Means and standard error of means (SEMs) were calculated and used for the presentation of data in figures. Data for relative gene expression, cytokine production, absolute cell counts, and CFSE data from flow cytometry analysis were compared with one-way ANOVA followed by Tukey multiple comparison tests for >3 comparisons. Differences between PBMC and PBL cultures were compared with paired *t*-tests. Differences were considered significant at *p* < 0.05.

## 5. Conclusions

**Figure 7:**
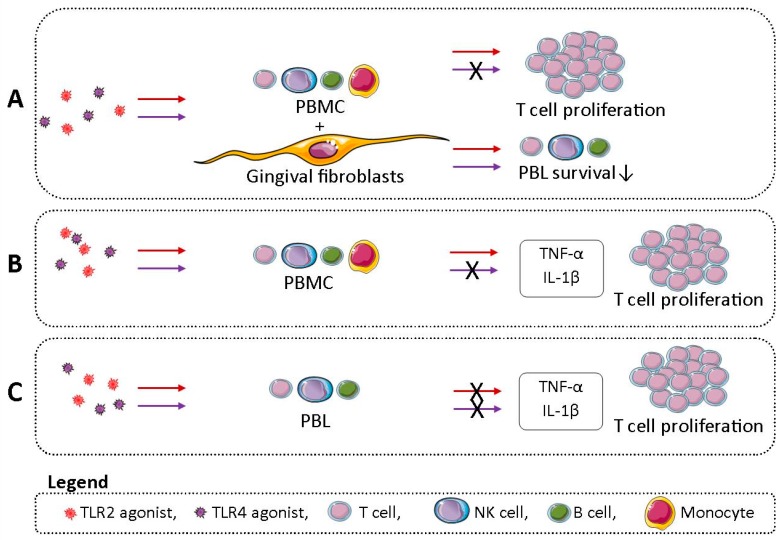
Graphical abstract illustrating the main findings of this study. (**A**) Chronic TLR2 (red arrow) and TLR4 (purple arrow) stimulation of cocultures of gingival fibroblast (GF) with peripheral blood mononuclear cells (PBMCs; T, NK, B cells, and monocytes) lead to reduced survival of peripheral blood lymphocytes (PBLs; T, NK, and B cells). TLR2 and not TLR4 stimulation of GF–PBMC cocultures lead to increased T cell proliferation. (**B**) TLR2 and not TLR4-stimulated PBMCs showed increased proinflammatory cytokine production of tumor necrosis alpha (TNF-α) and interleukin beta (IL-1β) and increased T cell proliferation. (**C**) TLR2 and/or TLR4 stimulation of PBL did not lead to TNF-α and IL-1β production or increased T cell proliferation.

The main findings of this study are summarized in the graphical abstract (Figure 7). In conclusion, GFs and monocytes mediate the diversity of cellular populations at the site of inflammation. First of all, GFs respond to the chronic challenge with TLR agonists by reducing the number of NK, B, and T cells. Moreover, TLR2 stimulation (and, to a lesser extent, TLR4 stimulation) of GF–PBMC or GF–PBL cocultures leads to increased T cell proliferation. Secondly, we demonstrate that TLR2 and not TLR4 activation plays an important role in T cell proliferation and proinflammatory cytokine production in cultures of PBLs with monocytes. This suggests that activation of TLR2 bridges innate and adaptive immunity.

## Figures and Tables

**Table 1:** Primer sequences used for quantitative PCR experiments.

Gene		Primer Sequence
*TLR2*	Forward Reverse	GGCTTCTCTGTCTTGTGACCG GAGCCCTGAGGGAATGGAG
*TLR4*	Forward Reverse	CTGCAATGGATCAAGGAACCAG CCATTCGTTCAACTTCCACCA
*KI67*	Forward Reverse	CGAGACGCCTGGTTACTATCAA GGATACGGATGTCACATTCAATACC
*HPRT*	Forward Reverse	TGACCTTGATTTATTTTGCATACC CGAGCAAGACGTTCAGTCCT

**Table 2:** Monoclonal antibodies used for flow cytometry experiments.

Antibody	Fluorochrome	Vendor/Cat No./Clone	Laser Lines	Emission Filters
Anti-human CD56	PE	eBiosciences by Thermo Fisher Scientific/#12056742/MSSB	488 nm	586/42
Anti-human CD3	BV506	eBiosciences by Thermo Fisher Scientific/#563109/UCHT1	405 nm	528/45
Anti-human CD19	APC	eBiosciences by Thermo Fisher Scientific/#17019842/SJ25C1	640 nm	660/10
Anti-human CD14	Efluor 450	BD biosciences /#48014942/61D3	405 nm	448/45

## Supplementary Materials

Supplementary materials can be found at https://www.mdpi.com/1422-0067/20/24/6134/s1.

## Abbreviations

CD	cluster of differentiation
CFSE	carboxyfluorescein succinimidyl ester
Ct	cycle threshold
ELISA	enzyme-linked immunosorbent assay
FCS	fetal calf serum
GF	gingival fibroblast
*HPRT*	*hypoxanthine phosphoribosyltransferase*
IL-1β	interleukin 1 beta
LPS	lipopolysaccharide
PBL	peripheral blood lymphocyte
PBMC	peripheral blood mononuclear cell
*Pg*	*Porphyromonas gingivalis*
TLR	toll-like receptor
TNF-α	tumor necrosis alpha
