# Supplementary material for: T Cell Proliferation Is Induced by Chronically TLR2-Stimulated Gingival Fibroblasts or Monocytes
Source: Int J Mol Sci. 2019 Dec 5;20(24):6134. doi: 10.3390/ijms20246134 (PMC6940768; doi:10.3390/ijms20246134)
Supplement: Supplementary file 1 [file ijms-20-06134-s001.pdf]

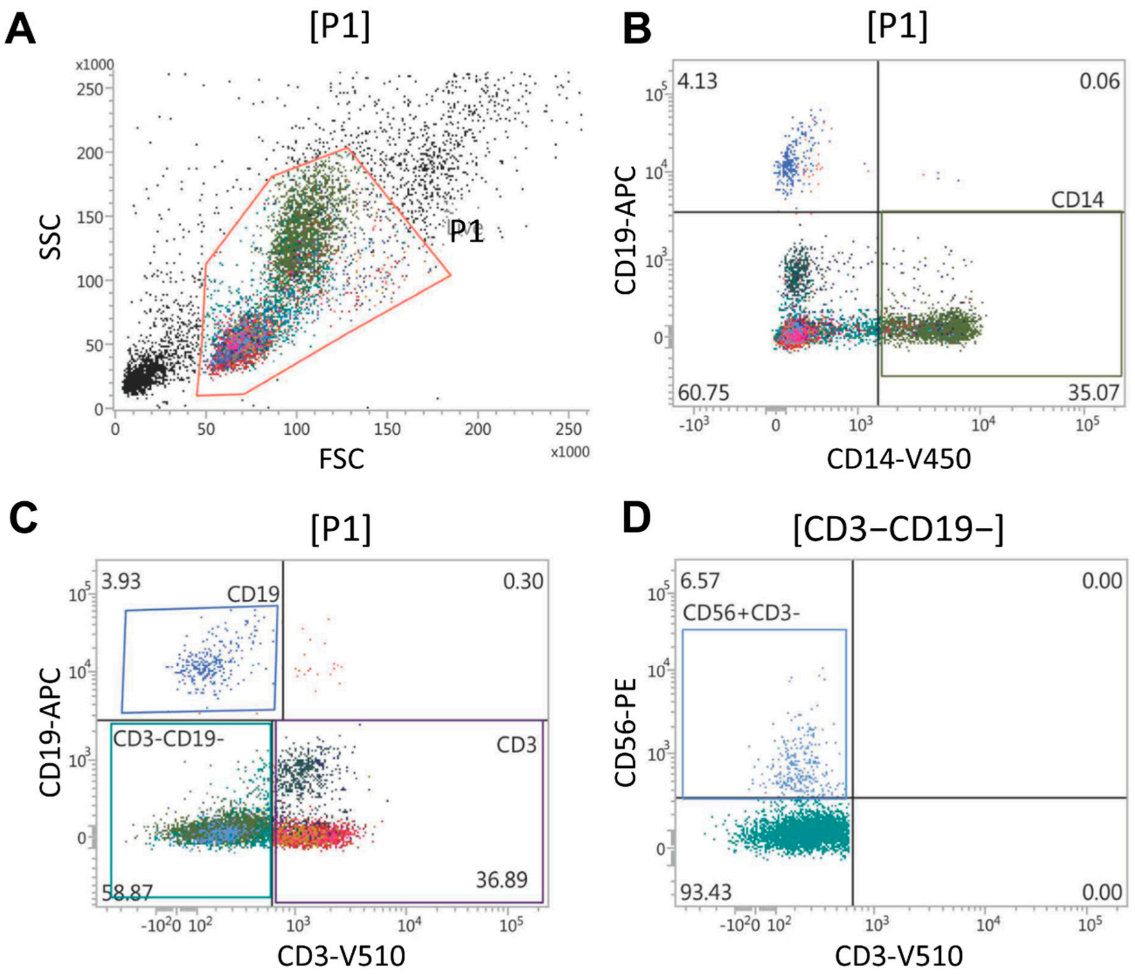

Supplementary Figure S1: Gating strategy. The antibody combination for flow cytometric experiments was CD19-APC/CD14-efluor450/CD3-BV510/CD14-PerCP-Cy5.5/CD56-PE (enlisted in Table 1). The number in quadrants present percentages of cells in the corresponding gate. The gating tree was set as follows: (A) forward scatter (FSC)/ sideward scatter (SSC) representing the cell distribution based on size and intracellular complexity (gate P1), respectively. Gate P1is encircled in red. In the [P1] population, (B) CD14+ (monocytes), (C) CD19+ (B-cells), and CD3+ (T-cells), and CD3−CD19− cells were identified. In the [CD3−CD19−] population, (D) CD56+CD3− cells (NK cells) were identified.

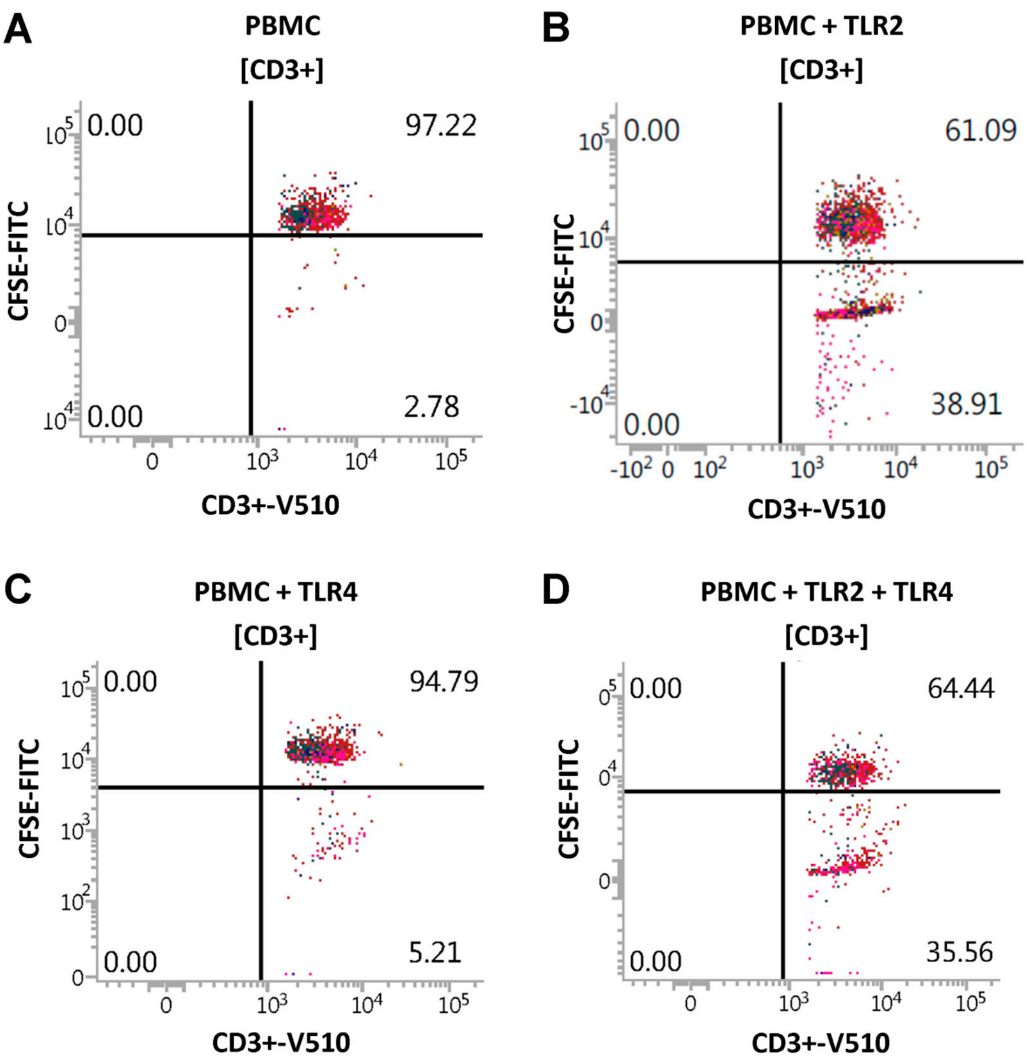

Supplementary Figure S2: Gating strategy of proliferation data. Quantification of the proliferation of carboxyfluorescein succinimidyl ester (CFSE)-labeled PBMCs after 14 days. Since only CD3+ (T-cells) proliferated, the proliferation of CD3+ is shown in all plots. Here, CFSE intensity and CD3 is plotted on the y-axis and x-axis, respectively. Proliferation by means of a demarcated CFSE population with lower fluorescence indicates the percentage of cells that have divided. Accordingly, the number in lower quadrants present percentages of cells that have divided. Proliferation was observed for cultures of (A) PBMCs, (B) PBMCs cultured with TLR2, (C) PBMCs cultured with TLR4 and (D) PBMCs cultured with TLR2 and TLR4. Quantification of the divided CD3+ cells is shown in Figure 4D-F of this manuscript.

Supplementary Table S1: Proliferation data of CD19+, CD56+CD3-, and CD14+ cells. Quantification of the proliferation of carboxyfluorescein succinimidyl ester (CFSE)-labeled PBMCs and PBLs after 7 and 14 and 21 days. Data is presented as mean percentage proliferation ± standard deviation. n = 3.

Culture condition	Time (days)	CD19+CFSE- (PBMC)	CD19+CFSE- (PBL)	CD14+CFSE-	CD56+CD3-
Monocultures	t = 7	2.38 ± 1.99	0.77 ± 0.63	Not detectable (0.00 ± 0.00)	Not detectable (0.00 ± 0.00)
	t = 14	3.82 ± 1.41	0.50 ± 0.56	Not detectable (0.00 ± 0.00)	Not detectable (0.00 ± 0.00)
	t = 21	1.11 ± 1.92	Not detectable (0.00 ± 0.00)	Not detectable (0.00 ± 0.00)	Not detectable (0.00 ± 0.00)
TLR2	t = 7	2.47 ± 1.38	1.11 ± 0.91	Not detectable (0.00 ± 0.00)	Not detectable (0.00 ± 0.00)
	t = 14	3.90 ± 2.31	0.40 ± 0.21	Not detectable (0.00 ± 0.00)	Not detectable (0.00 ± 0.00)
	t = 21	3.19 ± 2.97	Not detectable (0.00 ± 0.00)	Not detectable (0.00 ± 0.00)	Not detectable (0.00 ± 0.00)
TLR4	t = 7	1.17 ± 0.61	0.92 ± 0.63	Not detectable (0.00 ± 0.00)	Not detectable (0.00 ± 0.00)
	t = 14	2.08 ± 1.34	1.11 ± 1.17	Not detectable (0.00 ± 0.00)	Not detectable (0.00 ± 0.00)
	t = 21	1.24 ± 1.22	1.19 ± 2.06	Not detectable (0.00 ± 0.00)	Not detectable (0.00 ± 0.00)
TLR2 + TLR4	t = 7	1.02 ± 0.46	0.37 ± 0.36	Not detectable (0.00 ± 0.00)	Not detectable (0.00 ± 0.00)
	t = 14	5.97 ± 0.75	1.15 ± 0.21	Not detectable (0.00 ± 0.00)	Not detectable (0.00 ± 0.00)
	t = 21	1.67 ± 2.89	1.98 ± 0.49	Not detectable (0.00 ± 0.00)	Not detectable (0.00 ± 0.00)
